# Maternal Antibody Transfer of a Fiber-Based Subunit Vaccine Confers Complete Protection Against Fowl Adenovirus Serotype 11 in Neonatal Chickens

**DOI:** 10.3390/vetsci13090928

**Published:** 2026-09-09

**Authors:** Guopan Li, Sirong Gao, Xiaoyang Ai, Jun Rong, Disi Chen, Lianshen Cai, Weiwei Guo

**Affiliations:** 1 College of Life Sciences, Yangtze University, Jingzhou 434000, China; 501155@yangtzeu.edu.cn (G.L.); 19972656574@163.com (S.G.); chendisi_262@163.com (D.C.); 2 Qingdao Yebio Bioengineering Co., Ltd., Qingdao 266000, China; cls_yb@126.com (L.C.); gwwgpp@126.com (W.G.)

**Keywords:** fowl adenovirus serotype 11, fiber protein, subunit vaccine, maternal antibody, passive immunity, inclusion body hepatitis

## Abstract

Fowl adenovirus serotype 11 (FAdV-11) is an emerging pathogen associated with inclusion body hepatitis (IBH) and high mortality in commercial broilers. In this study, we evaluated the protective efficacy of maternal antibody transfer against FAdV-11 in neonatal chicks. A hypervirulent FAdV-11 strain (FAdV-11/HB2022/1), isolated from an IBH outbreak in Hubei Province, China, caused 90% mortality in 1-day-old specific-pathogen-free (SPF) chickens; 7-day-old chicks exhibited no mortality but developed mild hepatic lesions and sustained viral replication. A soluble, trimeric Fiber subunit vaccine (Fiber_282__–__569_) was developed through rational truncation of the transmembrane domain, retaining both the shaft and head domains, with its homotrimeric conformation confirmed by size-exclusion chromatography coupled with multi-angle light scattering (SEC-MALS). Immunization of breeder hens with Fiber_282–569_ elicited high titers of FAdV-11-specific IgY and neutralizing antibodies that were efficiently transferred to progeny via the egg yolk. Upon lethal challenge with FAdV-11/HB2022/1, 1-day-old progeny chicks from vaccinated breeders exhibited 100% survival with no clinical signs, no gross pathological lesions, and undetectable viral loads in liver tissues, demonstrating complete protection. In contrast, all chicks from unvaccinated breeders succumbed within 5 days post-infection. These results demonstrate that maternal antibody transfer of the fiber-based subunit vaccine confers complete protection against lethal FAdV-11 infection in neonatal chicks, providing a safe and effective candidate strategy for controlling FAdV-11 in the poultry industry.

## Simple Summary

Highly virulent fowl adenovirus serotype 11 (FAdV-11) causes inclusion body hepatitis (IBH) and mass death in newly hatched broilers, generating severe economic losses for poultry farms worldwide. Young chicks under 7 days old are extremely susceptible to this virus, yet their immature immune systems cannot gain effective protection via routine active vaccination. In this work, we designed a truncated soluble trimeric Fiber antigen and verified its natural protein structure. Vaccinating breeder hens with this subunit vaccine induced high levels of protective antibodies that pass to offspring through egg yolk. After lethal virus exposure, all 1-day-old chicks carrying maternal antibodies survived with no disease signs, liver lesions or detectable virus in organs, while all unprotected control chicks died within five days. This breeder vaccination strategy delivers full passive protection to neonatal chicks under laboratory conditions, offering a promising candidate solution for FAdV-11 control in commercial poultry production pending further validation under commercial field conditions.

## 1. Introduction

Fowl adenoviruses (FAdVs) are non-enveloped, double-stranded DNA viruses belonging to the genus Aviadenovirus within the family Adenoviridae. Based on antigenic characteristics, FAdVs are classified into five species (FAdV-A through FAdV-E), encompassing at least 12 recognized serotypes [1,2]. Among these, FAdV-11, a member of species D, has emerged as a significant pathogen associated with inclusion body hepatitis (IBH); this disease primarily affects young chicks, while adult chickens typically exhibit subclinical infections [3].

Historically, FAdV-11 was considered to have low pathogenicity and was often isolated from clinically healthy flocks. However, since approximately 2015, an increasing number of severe IBH outbreaks linked to FAdV-11 have been reported globally, particularly in China, where mortality rates in affected flocks have reached 20–80% [4,5]. Phylogenetic analyses suggest the emergence and widespread dissemination of a hypervirulent lineage, yet the pathogenic mechanisms underlying this increased virulence remain poorly understood, and effective vaccines are still lacking.

The development of an effective vaccine against FAdV-11 is complicated by several factors, and the unique properties of this serotype make separate, targeted immunization necessary. First, FAdVs exhibit considerable genetic diversity, and cross-protection between serotypes is limited. As a member of FAdV species D, FAdV-11 differs substantially from the more widely studied FAdV-4 (species C) and FAdV-8 (species E) in the amino acid sequence of the Fiber protein—the major target of neutralizing antibodies. This divergence in key protective epitopes means that existing vaccines developed for FAdV-4 or FAdV-8 cannot provide effective cross-protection against FAdV-11, necessitating serotype-specific vaccine development [6]. Second, the clinical manifestation of FAdV-11 infection is strongly age-dependent: neonatal chicks (1–7 days old) are highly susceptible with high pathogenicity, whereas older chickens (>14 days) are largely resistant [3,7]; even if subclinically infected, their pathogenicity is significantly attenuated compared to chicks, likely due to immune system maturation. This age-dependent susceptibility poses a significant challenge for vaccination strategies. Beyond the immunological immaturity of neonatal chicks, establishing conventional active immunity requires at least 14 days [8], by which time chickens are often less susceptible to FAdV-11 with lower pathogenicity, making vaccine efficacy evaluation extremely difficult. Third, no commercially available vaccine currently targets FAdV-11 specifically. While live attenuated and inactivated vaccines exist for other FAdV serotypes such as FAdV-4 [9], their limited cross-reactivity with FAdV-11 restricts their utility against this pathogen. Compounding this, historically low-pathogenic FAdV-11 has evolved into hypervirulent lineages in recent years, causing high-mortality IBH outbreaks worldwide and creating an urgent unmet need for targeted immunization [6].

Subunit vaccines based on recombinant viral proteins offer a safe, cost-effective, efficient, and environmentally friendly alternative. The fiber protein is a key determinant of viral attachment and cell tropism, making it an ideal vaccine candidate [4,10,11,12]. The native fiber protein exists as a homotrimer, and this conformation is essential for eliciting potent neutralizing antibody responses. Previous studies on FAdV-4 and FAdV-8 have demonstrated the efficacy of fiber-based subunit vaccines [13,14]. However, obtaining soluble and properly folded trimeric fiber proteins via *Escherichia coli* expression systems—especially those derived from FAdV-11—remains a major challenge. The hydrophobic transmembrane domain readily causes protein aggregation in prokaryotic expression systems, thereby compromising the immunogenicity of the antigen.

An alternative strategy to overcome the challenge of age-dependent susceptibility is to exploit maternal antibody-mediated passive immunity [15,16]. In poultry, immunoglobulin Y (IgY) is efficiently transferred from the hen to the egg yolk, providing progeny with immediate protection during their most vulnerable early days of life. This “breeder vaccination–progeny protection” strategy has been successfully employed for controlling several avian diseases [13,17], but its application to FAdV-11 has not been systematically investigated.

In this study, we isolated a virulent FAdV-11 strain (FAdV-11/HB2022/1) and characterized its pathogenicity in SPF chickens. Subsequently, we developed a soluble trimeric fiber subunit vaccine (Fiber_282–569_) using a rational truncation strategy and validated its trimeric conformation via size-exclusion chromatography coupled with multi-angle light scattering (SEC-MALS). We established a “breeder hen immunization–progeny challenge” model to evaluate whether maternal antibodies could confer protection against lethal FAdV-11 infection in neonatal chicks. Our results demonstrate that this strategy confers complete clinical protection and undetectable hepatic viral loads in neonatal chicks under lethal challenge, establishing a promising new approach for controlling FAdV-11.

## 2. Materials and Methods

### 2.1. Ethics Statement

Research Animal experimentation complied with the national standard GB/T 35892-2018 (Guidelines for Ethical Review of Laboratory Animal Welfare) and received approval from the Yangtze University Animal Ethics Committee (approval No. 2021-01-002, approval date: 5 January 2021). All procedures adhered to the 3R principles. Strict humane endpoints were enforced during challenge studies; birds displaying severe lethargy, anorexia exceeding 12 h, or loss of ambulation were euthanized immediately.

All animal experiments were implemented following rigorous experimental design norms. Randomization protocols for animal allocation in each assay are detailed in the corresponding experimental subsections. To minimize subjective assessment bias, investigators performing daily clinical observation, gross lesion evaluation, histopathological evaluation, and semi-quantitative pathological analysis were blinded to treatment group assignments throughout the entire study.

### 2.2. Clinical Specimens, Experimental Animals, and Cells

In March 2021, a flock of 3-day-old chicks in Hubei Province experienced acute mortality (~15%). Necropsy of affected birds revealed hepatomegaly with hemorrhage and necrosis. Liver tissues from five mortalities were harvested aseptically, minced, and homogenized (1:5 *w*/*v*) in PBS (pH 7.2) supplemented with 1% penicillin–streptomycin using a Servicebio high-throughput grinder (50 Hz, 2 min). Following three freeze–thaw cycles, the suspension was centrifuged (8000× *g*, 10 min) and filtered (0.22 μm) to generate a stock inoculum. While initial screening relied on PCR, histopathological confirmation was systematically performed in the subsequent pathogenicity experiments to characterize the lesions.

Specific pathogen-free (SPF) chicken embryos and 140-day-old SPF chickens were purchased from Xinxing Dahuanong Poultry Egg Co., Ltd. (Yunfu, China). Embryos were incubated in a forced-draft incubator. SPF chickens were reared in negative-pressure isolators. Newly hatched chicks (1–7 days old) were maintained at 35 ± 3 °C under an ambient temperature of 25 ± 2 °C and a relative humidity of 55% ± 10%. Sterilized water and an SPF diet were provided.

Chicken hepatocellular carcinoma (LMH) cells, maintained in our laboratory, were cultured in DMEM/F12 medium supplemented with 10% fetal bovine serum (FBS, Gibco, Grand Island, NY, USA), 100 U/mL penicillin, and 100 μg/mL streptomycin at 37 °C in a 5% CO_2_ incubator. Cells were subcultured at a 1:3 ratio every 3 days, and those in the logarithmic growth phase were used for experiments.

### 2.3. Phylogenetic Analysis

Total DNA was extracted from the aforementioned tissue homogenates using a commercial DNA extraction kit (Vazyme Biotech, Nanjing, China). Two pairs of primers were synthesized for PCR amplification: FAdV Hexon F1 (5′-TGGACATGGGGGCGACCTA-3′)/FAdV Hexon R1 (5′-TTGCCTGTGGCGAAAGGCG-3′) for amplification of the hexon gene, and FAdV Fiber F1 (5′-ATGGCAAAATCGACTCCTTTC-3′)/FAdV Fiber R1 (5′-GGGTTGTGTTAATTTATTGG-3′) for amplification of the fiber gene. PCR amplification conditions: initial denaturation at 95 °C for 5 min; 35 cycles of 95 °C for 30 s, 60 °C for 30 s, 72 °C for 1 min; final extension at 72 °C for 10 min. Amplicons were cloned into pTOPO-T vectors and verified by Sanger sequencing.

Phylogenetic analysis included 29 GenBank reference hexon sequences (Table S1) spanning FAdV species A–E. Sequences were aligned via ClustalW, and a neighbor-joining tree was constructed in MEGA 11.0 using the p-distance model with 1000 bootstrap replicates.

### 2.4. Virus Isolation of FAdV-11/HB2022/1

The PCR-confirmed FAdV-11-positive stock suspension was inoculated onto LMH cell monolayers at a 10% (*v*/*v*) inoculum. After adsorption for 1 h at 37 °C, the inoculum was replaced with maintenance medium containing 2% FBS. Cells were incubated for 3–5 days and observed daily for cytopathic effect (CPE). When CPE exceeded 75%, the cell suspension was harvested and subjected to three freeze–thaw cycles to release the virus. The viral stock was blindly passaged for three generations. Total DNA from third-passage infected cells was extracted and confirmed by PCR using the same primers. The positive viral isolate was designated FAdV-11/HB2022/1.

### 2.5. Indirect Immunofluorescence Assay (IFA)

LMH cells on coverslips (70% confluence) were infected with FAdV-11/HB2022/1 (MOI = 1); uninfected cells served as controls. At 24 h post-infection, cells were washed (PBS ×3), fixed (4% paraformaldehyde, 15 min, RT), permeabilized (0.1% Triton X-100, 10 min), and blocked (5% skim milk, 1 h, 37 °C). Samples were probed with laboratory-prepared FAdV-11-positive chicken serum (1:1000, 1 h, 37 °C). This serum was prepared by immunizing 21-day-old SPF chickens with formaldehyde-inactivated FAdV-11/HB2022/1 via intramuscular injection (two doses at a 2-week interval), with a confirmed neutralizing antibody titer of 2^9^ and verified specificity by IFA showing no cross-reactivity with FAdV-4. Samples were then incubated with FITC-conjugated rabbit anti-chicken IgY (Sigma-Aldrich, St. Louis, MO, USA; 1 h, 37 °C, dark). Coverslips were mounted with anti-fade medium and visualized using a fluorescence microscope (Olympus IX71, Tokyo, Japan).

### 2.6. Pathogenicity Assessment by Route and Age

To evaluate the pathogenicity of different inoculation routes, sixty 1-day-old SPF chicks were allocated into six groups (*n* = 10 per group) by stratified random sampling based on body weight, to ensure no significant difference in initial body weight across groups. Groups A–C received 10^7^ TCID_50_/bird of FAdV-11/HB2022/1 via intramuscular (IM), intraocular (IO), or intranasal (IN) routes, respectively; Groups D–F received PBS via corresponding routes. Birds were monitored for 7 days post-infection (dpi) for clinical signs, mortality, and body weight. Survivors were euthanized at 7 dpi for qPCR-based viral load quantification (liver, spleen, bursa, kidney) and hepatic histopathology (HE staining).

To evaluate the effect of age at challenge on pathogenicity, fifty 1-day-old SPF chicks were allocated to five groups (*n* = 10 per group) by stratified random sampling based on body weight: challenge at 1, 7, 14, or 21 days of age, plus a PBS control. Challenge groups received 10^7^ TCID_50_/bird IM; controls received PBS. Mortality and body weight were recorded daily through 7 dpi, after which survivors were necropsied for gross lesion scoring and HE-stained histopathological examination.

### 2.7. Prokaryotic Expression of Fiber Protein

Based on the fiber protein sequence of FAdV-11/HB2022/1 (PQ249639), homology modeling was performed using SWISS-MODEL to analyze structural domains. To achieve high-level soluble expression in *E. coli*, a truncated construct was designed by removing the N-terminal hydrophobic transmembrane and cytoplasmic regions (aa 1–281) while retaining the shaft domain (aa 282–355, critical for trimerization) and the head domain (aa 361–569, containing major epitopes), connected by a (Gly_4_Ser)_3_ linker. The codon-optimized gene was synthesized (Sangon Biotech, Shanghai, China) and cloned into pET-28a(+) to generate the recombinant plasmid pET28a-Fiber_282–569_, which was then transformed into *E. coli* BL21(DE3) competent cells.

Positive clones were cultured in LB medium containing 50 μg/mL kanamycin at 37 °C to an OD_600_ of 0.6–0.8. Expression was induced with 30 mM α-lactose at 32 °C for 12 h. Cells were harvested, resuspended in PBS, and disrupted by ultrasonication. After centrifugation at 12000× *g* for 20 min at 4 °C, the supernatant was collected.

### 2.8. Purification of Recombinant Fiber_282–569_ Protein

The supernatant was fractionated by ammonium sulfate precipitation. Solid (NH_4_)_2_SO_4_ was added to 30% saturation, and after standing for 2 h at 4 °C, the precipitate was removed by centrifugation (12,000× *g*, 10 min). The supernatant was then brought to 40% saturation, held at 4 °C for another 2 h, and centrifuged to collect the precipitate. The pellet was redissolved in 0.02 M PB (pH 7.4) at a 1:5 (*w*/*v*) ratio and dialyzed extensively against 0.01 M PBS (pH 7.4) to equilibrate the buffer. The dialyzed solution was clarified by centrifugation and filtration and then purified using an AKTA Purifier 100 system (Cytiva, Marlborough, MA, USA) equipped with a Sepharose Ni-TA Fast Flow affinity column (Bestchrom, Shanghai, China) pre-equilibrated with 0.01 M PBS (pH 7.4). After sample loading, the column was washed with equilibration buffer, followed by wash buffer (0.01 M PBS, 10 mM imidazole, pH 7.4) to remove impurities. The target protein was eluted with elution buffer (0.01 M PBS, 50 mM imidazole, pH 7.4). Purity and solubility were assessed by SDS-PAGE, and protein concentration was determined by the BCA assay.

### 2.9. Western Blot

Purified Fiber_282–569_ and clinical tissue homogenates were resolved on 12% SDS-PAGE and transferred to PVDF membranes. Membranes were blocked (5% non-fat milk/TBST, 1 h, RT) and probed overnight at 4 °C with lab-prepared chicken anti-FAdV-11 Fiber polyclonal antibody (1:500). After washing, blots were incubated with HRP-rabbit anti-chicken IgY (KPL, Gaithersburg, MD, USA; 1:10,000, 2 h, RT) and developed using Super ECL reagent (Yeasen, Shanghai, China). The original Western blot images with molecular-weight markers are provided in Supplementary Figure S1.

### 2.10. SEC-MALS Molecular Mass Analysis

Absolute molecular mass was determined using an AKTA pure 25L system (Cytiva, MA, USA) coupled with a Wyatt DAWN MALS detector (Wyatt, Santa Barbara, CA, USA), equipped with a Superose 6 Increase 10/300 GL column (Cytiva, Uppsala, Sweden). Filtered sample (0.2 mL) was injected and eluted at 1.0 mL/min in 0.01 M PBS (pH 7.4, 0.1-μm filtered). Data were processed with ASTRA 7 software.

### 2.11. Subunit Vaccine Preparation, Immunization, and Challenge

Purified Fiber_282–569_ protein was filter-sterilized, diluted in PBS, and supplemented with 2% Tween-80 to form the aqueous phase. This was emulsified with Montanide ISA 71 VG (white oil adjuvant, purchased from SEPPIC, Paris, France) at a 1:3 (*v*/*v*, aqueous:oil) ratio using a high-speed shear homogenizer at 20,000 rpm for 15 min (intermittent) to yield a stable water-in-oil subunit vaccine containing 50 μg/mL antigen.

Twelve 140-day-old SPF hens and two SPF roosters were divided into an immunized group (6 hens + 2 roosters) and a control group (6 hens + 2 roosters). Each hen in the immunized group received 0.5 mL of the vaccine (25 μg antigen) intramuscularly in the breast muscle, with a booster of the same dose 2 weeks later; controls received PBS. Fiber-specific antibody levels were monitored by indirect ELISA, and neutralizing antibody titers were determined using a microneutralization assay in LMH cells. Starting at 4 weeks post-immunization, hatching eggs were collected from all 6 hens in each group and incubated under identical conditions to generate progeny pools: chicks from immunized breeders carried maternal antibodies, whereas chicks from control breeders did not. Total IgY and neutralizing antibody titers were verified in 1-day-old chicks from each pool.

For the challenge experiment, a two-step random allocation strategy was adopted to balance maternal sources. First, 3–4 one-day-old chicks were randomly sampled from each of the 6 breeder hens in each group to form a progeny pool of 20 chicks, ensuring balanced representation of all maternal sources. Second, chicks in each progeny pool were allocated into two subgroups (*n* = 10 per subgroup) by simple random sampling, with balanced distribution of offspring from each hen between subgroups: for immunized progeny, subgroup A (virus challenge) and subgroup B (PBS control); for control progeny, subgroup C (virus challenge) and subgroup D (PBS control). In this design, the individual chick was defined as the observational unit. Given the uniform SPF genetic background of all breeder hens and balanced sampling across hens, hen-level clustering effects on maternal antibody levels were considered minimal. Groups A and C were intramuscularly inoculated with 10^7^ TCID_50_ of FAdV-11/HB2022/1 per bird in a volume of 0.2 mL, while groups B and D received an equivalent volume of PBS via the same route. All birds were observed for 7 days post-infection (dpi), during which survival rates and body weight changes were recorded daily. At 7 dpi, all surviving birds were euthanized and subjected to necropsy. Liver tissues were collected for histopathological analysis by hematoxylin and eosin (HE) staining and for viral load determination by absolute quantitative PCR.

### 2.12. Viral Load Quantification by qPCR

A pair of qPCR primers, FAdV-11 RT F1 (5′-GGTTACAGACAAGCATTCAGG-3′) and FAdV-11 RT R1 (5′-TCAGAGACAGACCGTTAGATGA-3′), was designed based on the FAdV-11/HB2022/1 genome. Absolute quantitative PCR was performed as described previously [18], with each sample run in triplicate. Results are expressed as the geometric mean ± SD of DNA copies per milligram of liver tissue.

### 2.13. Indirect ELISA for Total IgY Antibody Titer Determination

Microtiter plates were coated overnight at 4 °C with Fiber_282–569_ (1 μg/mL in carbonate buffer, pH 9.6). After blocking (5% milk/PBST, 1 h, 37 °C), serial two-fold serum dilutions (starting 1:100) were added and incubated (1 h, 37 °C). Bound antibodies were detected with HRP-goat anti-chicken IgY (1:20,000, 1 h, 37 °C) and TMB substrate (15 min, RT, dark). Reactions were stopped with 2 M H_2_SO_4_ and read at OD_450_. Titers were defined as the highest dilution with S/N > 2.1; values <10^2^ were considered negative.

### 2.14. Microneutralization Assay for Neutralizing Antibody Titer Determination

Heat-inactivated sera (56 °C, 30 min) were serially diluted twofold and mixed with 100 TCID_50_ of FAdV-11/HB2022/1 (1 h, 37 °C). Mixtures were inoculated onto LMH monolayers, adsorbed (1 h, 37 °C, 5% CO_2_), and overlaid with maintenance medium. CPE was scored over 5–7 days. Neutralizing titers were defined as the reciprocal of the highest dilution completely preventing CPE; titers <2^3^ were deemed negative.

### 2.15. Statistical Analysis

Data are presented as mean ± SD. Animal challenge studies were performed as single biological experiments, with exact sample sizes (number of animals per group) specified in each experimental section and corresponding figure legends. Quantitative laboratory assays (qPCR, ELISA, and microneutralization assay) were performed in technical triplicates for each sample.

Survival curves were generated using the Kaplan–Meier method. Single-time-point endpoint body weight data were analyzed using ordinary one-way ANOVA followed by Tukey’s HSD post-hoc test. Longitudinal repeated-measures body weight data were analyzed using two-way mixed repeated-measures ANOVA to assess the main effects of treatment group and time, as well as the group × time interaction effect; Tukey’s HSD post-hoc test was used for multiple pairwise comparisons when significant main effects or interactions were detected. Antibody titers, viral load data, and organ index data were analyzed using ordinary one-way ANOVA followed by Tukey’s HSD post-hoc test. All statistical analyses were performed using GraphPad Prism 9.0. Significance thresholds were defined as: * *p* < 0.05, ** *p* < 0.01, *** *p* < 0.001; ns = not significant.

## 3. Results

### 3.1. Isolation and Identification of Strain FAdV-11/HB2022/1

**Figure 1:**
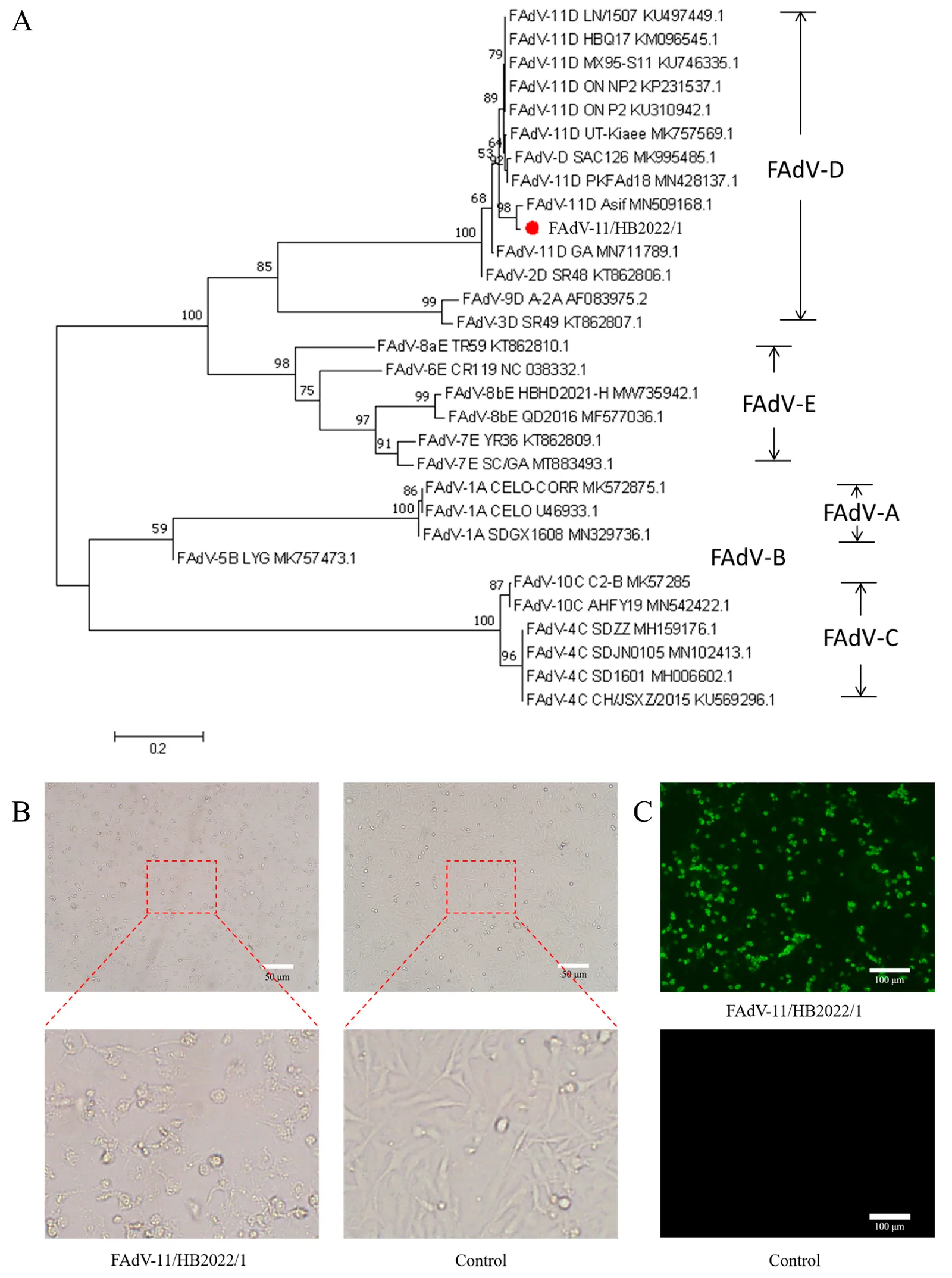
Isolation and identification of the FAdV-11/HB2022/1 strain. (**A**) Evolutionary tree analysis based on the partial hexon gene; 29 reference sequences spanning FAdV species A–E; neighbor-joining method and the p-distance model with 1000 bootstrap replicates. Red dots indicate the isolated strains.(**B**) CPE induced by the 3rd passage (P3) of FAdV-11/HB2022/1 in LMH cells at 48 h post-infection; scale bar: 50 μm. (**C**) IFA of FAdV-11-infected LMH cells; scale bar: 100 μm.

The hexon gene fragment (accession no. ON959238) encompassing the L1, L2, and L3 hypervariable regions and the fiber gene fragment (accession no. PQ249639) were successfully amplified from the clinical samples. Phylogenetic analysis based on the hexon gene sequences showed that the FAdV-11/HB2022/1 strain clustered with all reference FAdV-11 strains with 100% bootstrap support, forming a clade clearly separated from those of other serotypes (Figure 1A). The isolate was therefore identified as serotype FAdV-11.

When the tissue homogenate was inoculated onto LMH cells, no obvious cytopathic effect (CPE) was observed in the first passage. In the second passage, mild cell rounding appeared at 72 h post-inoculation, and in the third passage, marked CPE developed by 48 h, characterized by cell rounding, clustering, increased refractivity, partial detachment, and basophilic intranuclear inclusion bodies (Figure 1B). Indirect immunofluorescence assay (IFA) revealed specific green fluorescence in the nuclei of infected cells, whereas no fluorescence was observed in the negative control (Figure 1C), further confirming that the isolated strain is an FAdV-11 virus (species FAdV-D).

### 3.2. FAdV-11/HB2022/1 Is Highly Pathogenic via the Intramuscular Route

**Figure 2:**
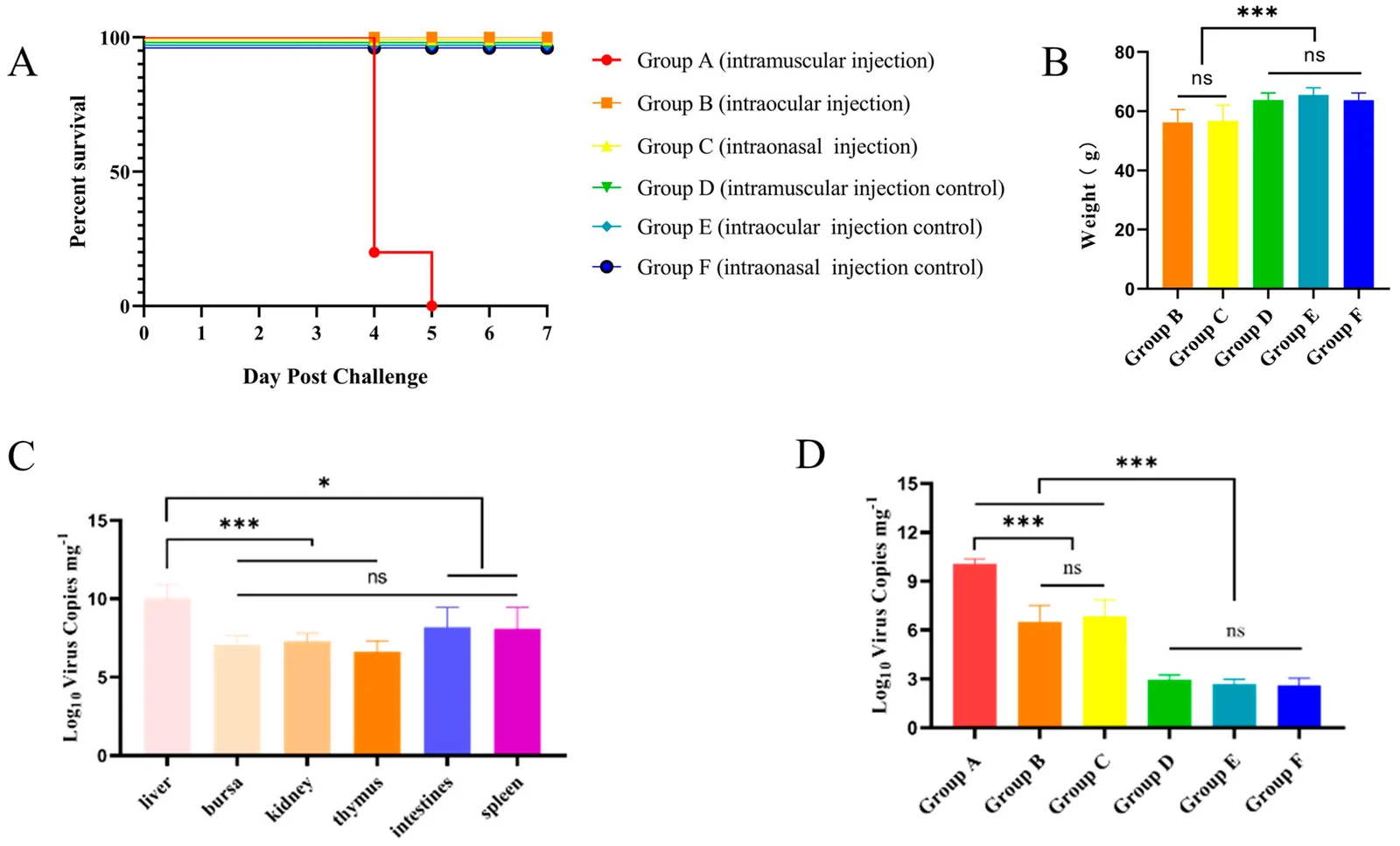
Pathogenicity of FAdV-11/HB2022/1 in 1-day-old SPF chicks via different inoculation routes. (**A**) Kaplan–Meier survival curves of chicks over 7 days post-infection (dpi). (**B**) Body weight of chicks at 7 dpi. Note: No body weight data are available for the intramuscular challenge group at 7 dpi, as all animals died prior to this time point. (**C**) Viral loads in different tissues from chicks that died following intramuscular challenge, quantified by absolute qPCR. (**D**) Hepatic viral loads at 7 dpi across all inoculation groups. Group definitions: Group A, intramuscular challenge with 10^7^ TCID_50_ FAdV-11/HB2022/1; Group B, intraocular challenge with 10^7^ TCID_50_ FAdV-11/HB2022/1; Group C, intranasal challenge with 10^7^ TCID_50_ FAdV-11/HB2022/1; Group D, intramuscular PBS control; Group E, intraocular PBS control; Group F, intranasal PBS control. *n* = 10 chicks per group. Data are presented as mean ± SD. * *p* < 0.05, *** *p* < 0.001, ns = not significant.

Following intramuscular challenge of 1-day-old chicks, depression, anorexia, and diarrhea appeared on day 2. Eight chicks died on day 4 and the remaining two on day 5, resulting in 100% mortality with a mean death time of 4.2 days (Figure 2A). Body weight was significantly lower than that of the control group (Figure 2B). The virus was mainly detected in the liver; it was also distributed in the bursa of Fabricius, kidneys, thymus, spleen, and small intestine, but with viral loads significantly lower than those in the liver (Figure 2C). In the intraocular and intranasal groups, no mortality occurred, but the birds showed depression, reduced feed intake, and slightly decreased body weight. The liver viral loads in these two groups were significantly higher than that of the control group but lower than that of the intramuscular challenge group (Figure 2D). These results indicate that FAdV-11/HB2022/1 exhibits the strongest pathogenicity in chicks via intramuscular injection, while mucosal infection leads to attenuated virulence.

### 3.3. FAdV-11/HB2022/1 Is Highly Susceptible and Pathogenic to Young SPF Chicks

**Figure 3:**
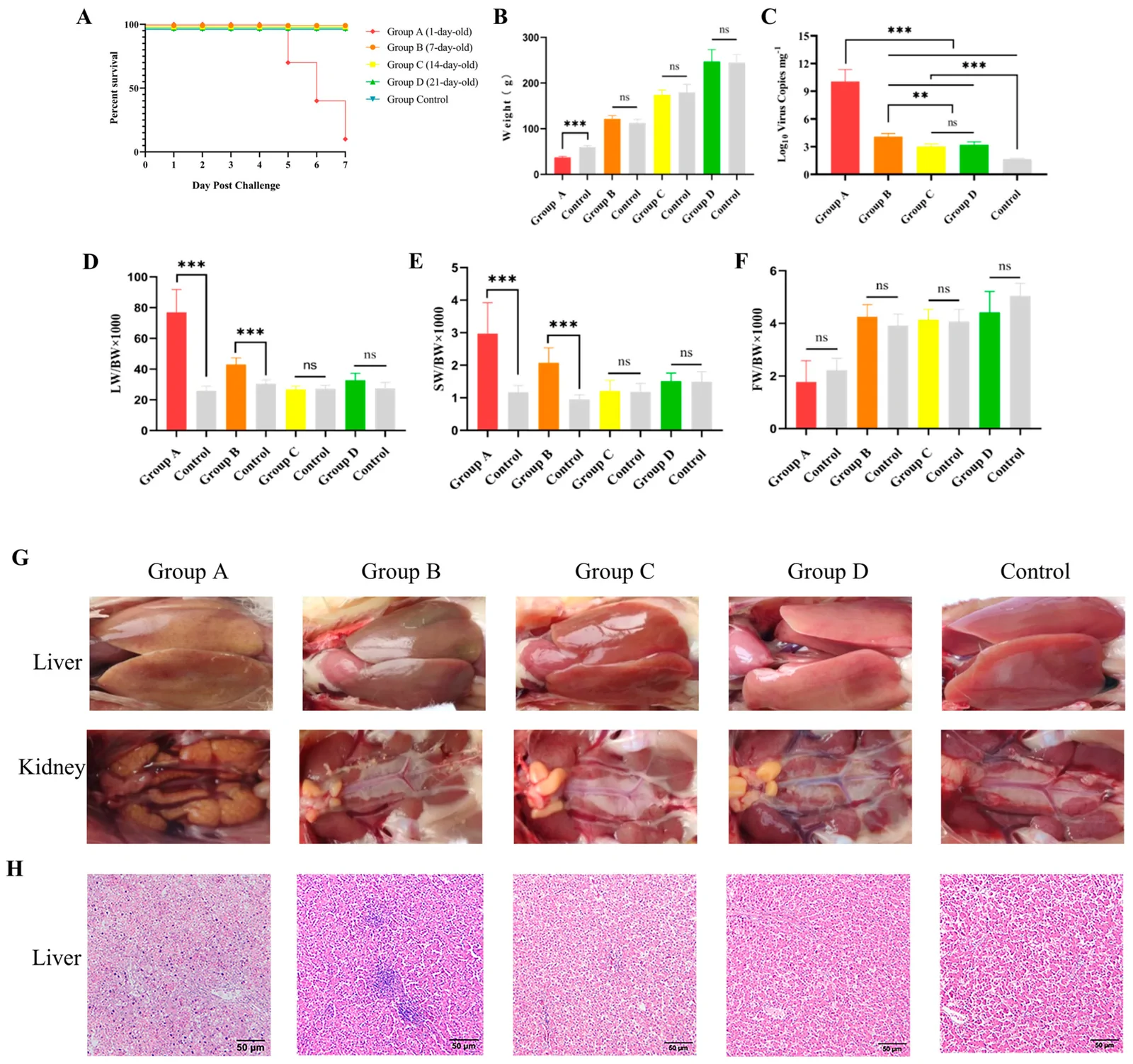
Age-dependent pathogenicity of FAdV-11/HB2022/1 in SPF chickens. (**A**) Kaplan–Meier survival curves over 7 dpi for chickens challenged at different ages. (**B**) Body weight changes of chickens at 7 dpi. Note: All chicks in the intramuscular challenge group (Group A) succumbed to infection prior to 7 dpi; the body weight value presented for Group A is derived from the last recorded body weight of surviving animals before death, instead of the actual measurement at 7 dpi. (**C**) Hepatic viral loads at 7 dpi for chickens challenged at different ages, quantified by absolute qPCR. (**D**–**F**) Organ index (organ weight/body weight × 1000) of liver (**D**), spleen (**E**) and bursa of Fabricius (**F**) at 7 dpi. (**G**) Gross pathological changes of liver and kidney at 7 dpi in each group. (**H**) Histopathological changes of liver tissues at 7 dpi (HE staining). Group definitions: Group A, challenged at 1 day of age; Group B, challenged at 7 days of age; Group C, challenged at 14 days of age; Group D, challenged at 21 days of age; Control, age-matched PBS control. All challenge groups received 10^7^ TCID_50_ FAdV-11/HB2022/1 via intramuscular injection. *n* = 10 chicks per group. Data are presented as mean ± SD. ** *p* < 0.01, *** *p* < 0.001, ns = not significant. Scale bar: 50 μm.

The challenge outcomes varied markedly with the age of the birds. The survival curve showed that the group challenged at 1 day of age experienced 90% mortality (9/10), with a mean death time of 6 days (Figure 3A). The single surviving chick displayed severe hepatomegaly, hemorrhage, and necrosis (Figure 3G). In contrast, all chicks challenged at 7, 14, or 21 days of age survived. Body weight measurement demonstrated a significant decline in the 1-day-old challenge group, whereas growth was unaffected in the other challenge groups (Figure 3B). Viral load quantification revealed a close correlation with challenge age: the highest load was found in the 1-day-old challenge group, followed by the 7-day-old group; the viral loads in the 14- and 21-day-old groups were comparable, but significantly lower than those in the 1- and 7-day-old groups, while remaining higher than that of the control group (Figure 3C). To reflect the impact on organs, liver index (liver weight/body weight × 1000), spleen index (spleen weight/body weight × 1000), and bursa of Fabricius index (bursa weight/body weight × 1000) were used to indicate swelling or atrophy. Challenge at 1 and 7 days of age caused swelling of the liver (Figure 3D) and spleen (Figure 3E), whereas challenge at 14 and 21 days had no effect. The influence on bursal size was negligible across all challenge ages (Figure 3F). Necropsy revealed that 1-day-old challenged chicks developed severe inclusion body hepatitis (IBH) with swollen, hemorrhagic, yellowish, and friable livers, accompanied by mild renal swelling and pallor. Typical IBH lesions, albeit milder, were also observed in the livers of the 7-day-old challenge group, with no obvious gross abnormalities in the kidneys. Livers and kidneys from the 14- and 21-day-old challenge groups appeared grossly normal (Figure 3G). HE staining showed massive hepatocellular necrosis, numerous basophilic intranuclear inclusion bodies, severe mixed inflammatory cell infiltration in portal areas, and abundant lipid droplets in hepatocytes of the 1-day-old challenge group. Similar pathological changes, but to a lesser degree, were seen in the 7-day-old group. Only occasional inflammatory cell infiltration was observed in the 14- and 21-day-old challenge groups (Figure 3H).

### 3.4. Soluble Expression of Fiber_282–569_ Protein in E. coli

To address the issue of full-length Fiber protein (aa 1–569) being expressed as inclusion bodies in the prokaryotic system with low immunogenicity, homology modeling of Fiber_1–569_ was performed using SWISS-MODEL. The results showed that this sequence shares similarity with 7w83.1.A (FAdV-4 Fiber-2 protein) and 3s6x.1.A/6gap.1.A (outer capsid protein sigma-1) in different coverage regions (Table 1), suggesting that FAdV-11 Fiber can form a homotrimeric structure. The C-terminal region (aa 361–569) aligned well with the FAdV-4 Fiber-2 structure, constituting the “head” domain that contains the major epitopes for immune recognition and response. The N-terminal regions (aa 282–355, 97–161, 166–225, 320–359) exhibited varying degrees of similarity to the reovirus outer capsid attachment protein sigma-1, forming the “tail-shaft” that anchors the Fiber to the viral surface and stabilizes its structure.

**Figure 4:**
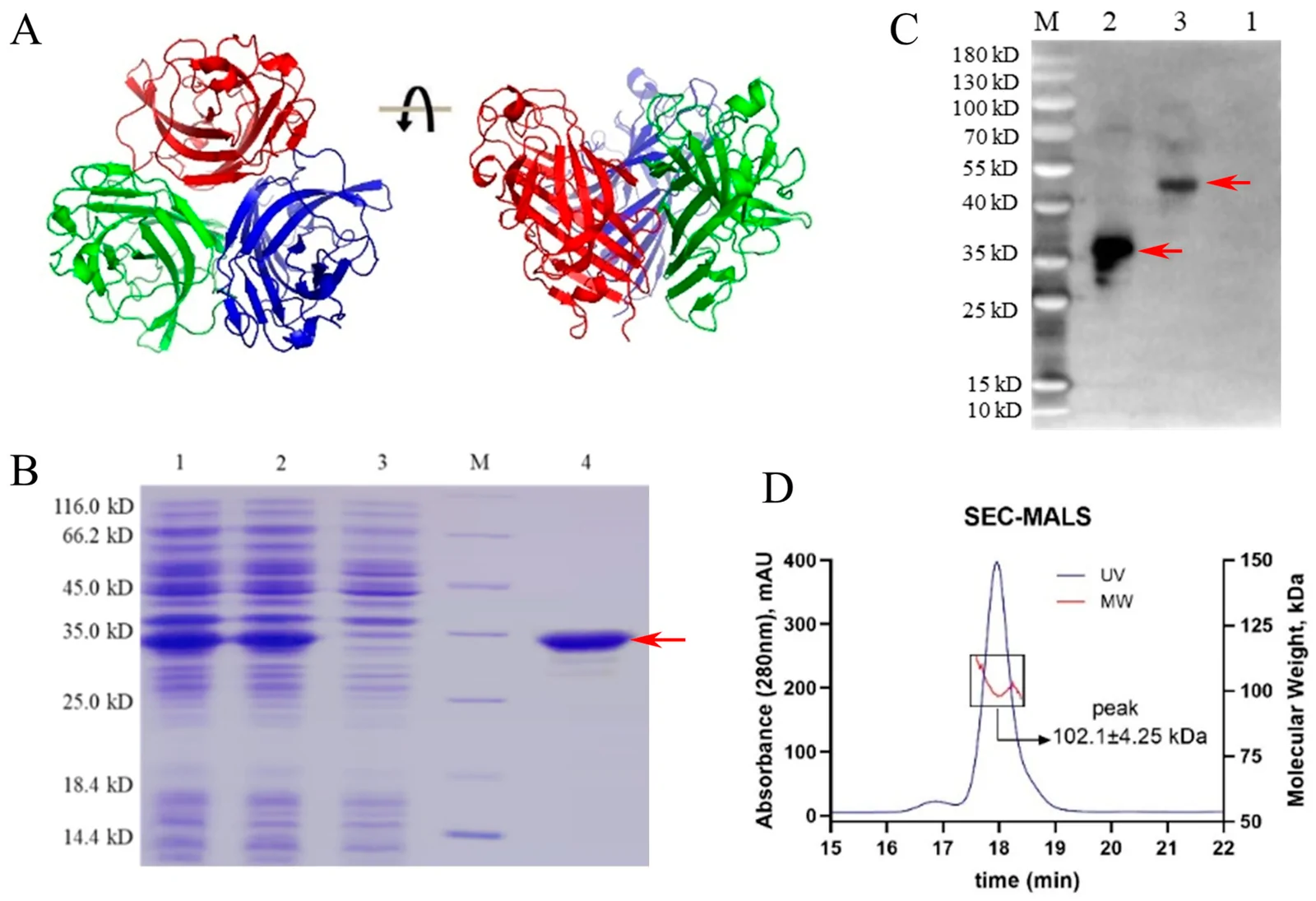
Expression, purification and identification results of Fiber_282–569_ in *E. coli*. (**A**) Predicted structure of Fiber_282–569_ obtained using SWISS-MODEL. Red, blue and green represent one subunit, respectively. (**B**) *E. coli* expresses soluble Fiber_282–569_. Lane 1: Total protein expressed by the *E. coli* strain of Fiber_282–569_; Lane 2: Soluble protein expressed by the *E. coli* strain of Fiber_282–569_; Lane 3: Negative control; M: Protein molecular weight standard; Lane 4: Purified Fiber_282–569_ protein. (**C**) Western blot results. Lane 1: Negative control; Lane 2: Purified Fiber_282–569_ protein; Lane 3: Positive sample tissue from FAdV-11/HB2022/1. (**D**) SEC-MALS measurement of absolute molecular weight. The blue curve is the ultraviolet absorption curve, and the red curve is the molecular weight distribution curve. The red arrow indicates the target protein band.

A construct was designed comprising the shaft domain (aa 282–355), which is critical for trimerization, and the head domain (aa 361–569), which harbors the major antigenic epitopes, connected by a (Gly_4_Ser)_3_ linker to generate a truncated Fiber_282–569_ protein. SWISS-MODEL prediction indicated that this truncated protein retains a trimeric conformation with an overall intact architecture (Figure 4A). Fiber_282–569_ was efficiently expressed in a soluble form in the *E. coli* BL21(DE3)/pET28a system, with a monomeric molecular mass of approximately 35 kDa, further suggesting correct folding. Following ammonium sulfate fractionation and affinity chromatography, the purity of Fiber_282–569_ was elevated to over 90% (Figure 4B). Western blot analysis confirmed that the truncated Fiber_282–569_ was specifically recognized by the FAdV-11 Fiber-positive antiserum, with a lower molecular mass than the full-length Fiber protein, as expected (Figure 4C). Size-exclusion chromatography coupled with multi-angle light scattering (SEC-MALS) revealed that the purified protein eluted as a major peak at t = 17.95 min, with an average apparent molecular mass of 102.1 ± 4.25 kDa (Figure 4D). The deviation from the theoretical trimer mass (106.2 kDa) was less than 5%, further confirming that Fiber_282–569_ forms a stable homotrimer.

### 3.5. Passive Immunity Conferred by Specific Anti-Fiber Maternal Antibodies Provides Complete Protection

**Figure 5:**
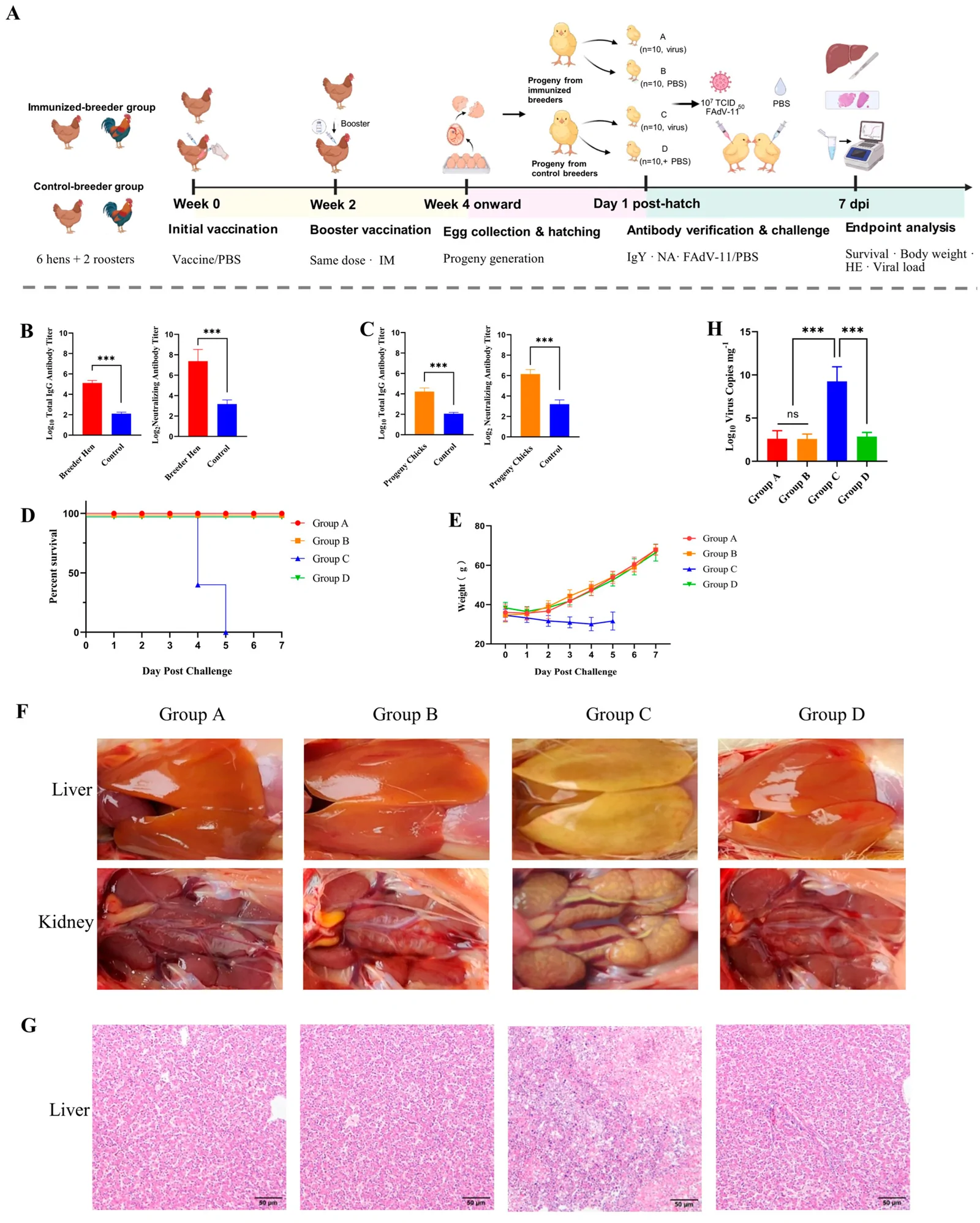
Protective efficacy of maternal antibodies induced by the fiber-based subunit vaccine against lethal FAdV-11 challenge in neonatal chicks. (**A**) Schedule of vaccination, hatch and challenge. In the schematic timelines, Week 0 represents the time of initial vaccination, Week 2 represents the time of booster vaccination, Week 4 represents the time of egg collection and hatching, day 1 post-hatch represents the time of challenge and antibody verification, and 7 dpi represents the time of endpoint analysis. (**B**) Fiber-specific total IgY titers and neutralizing antibody titers in immunized breeder hens, *n* = 6. (**C**) Fiber-specific total IgY titers and neutralizing antibody titers in 1-day-old progeny chicks, *n* = 20. (**D**) Kaplan–Meier survival curves of progeny chicks over 7 dpi, *n* = 10. (**E**) Body weight changes of progeny chicks over 7 dpi, *n* = 10; two-way mixed repeated-measures ANOVA with Tukey’s HSD post-hoc test. (**F**) Gross pathological examination of liver and spleen at 7 dpi. (**G**) Histopathological analysis of liver tissues at 7 dpi (HE staining, scale bar: 50 μm). (**H**) Hepatic viral loads at 7 dpi, quantified by absolute qPCR, *n* = 10. Data are presented as mean ± SD. *** *p* < 0.001. Group definitions: Group A, progeny from immunized breeders, challenged with FAdV-11/HB2022/1; Group B, progeny from immunized breeders, PBS control; Group C, progeny from control breeders, challenged with FAdV-11/HB2022/1; Group D, progeny from control breeders, PBS control. All challenge groups received 10^7^ TCID_50_ virus via intramuscular injection at 1 day of age.

Given the limited infectivity and pathogenicity of FAdV-11 in older SPF chickens, the conventional “immunization–challenge–evaluation” approach is not applicable for assessing FAdV-11 vaccine efficacy. Therefore, a “breeder hen immunization–progeny with maternal antibody–challenge–evaluation” strategy was adopted to evaluate the efficacy of the Fiber_282–569_ subunit vaccine. The overall experimental workflow and time schedule are summarized in Figure 5A: breeder hens received the primary immunization at Week 0, a booster immunization at Week 2; hatching eggs were collected at Week 4 and incubated; 1-day-old progeny chicks were subjected to antibody titer verification and lethal virus challenge; endpoint necropsy and sample analysis were performed at 7 days post-infection (dpi).

Two weeks after booster immunization, the serum Fiber-specific total IgY antibody titer in the immunized hens reached 10^5.11±0.25^, and the neutralizing antibody titer was 2^8.33±0.46^ (Figure 5B). Their 1-day-old progeny chicks carried high levels of maternal antibodies, with a Fiber-specific total IgY antibody titer of 10^4.23±0.35^ and a neutralizing antibody titer of 2^6.15±0.44^ (Figure 5C). All antibody levels in the progeny chicks from the control group were negative.

One-day-old progeny chicks were intramuscularly challenged with FAdV-11/HB2022/1. In the challenged control group, 100% of the chicks developed clinical disease. Mass mortality began on day 4 post-infection (dpi) (6/10), and all birds died by day 5 dpi, resulting in a 100% mortality rate with a mean time to death of 4.4 days (Figure 5D). Affected chicks exhibited significant body weight loss (Figure 5E). Necropsy and HE staining (Figure 5F,G) revealed severe hepatic hemorrhage and necrosis, with numerous intranuclear basophilic inclusion bodies observed in hepatocytes. Severe mixed inflammatory cell infiltration was present in the portal tracts, consistent with typical inclusion body hepatitis (IBH). Viral load determination also confirmed high viral titers in liver tissues (Figure 5H).

In contrast, progeny chicks with maternal antibodies remained clinically normal and showed no observable clinical signs following challenge, with normal feed intake and progressive body weight gain. Necropsy revealed that the liver and kidneys were normal in size, color, and texture. HE staining showed no pathological changes in the liver, and no FAdV-11 virus was detected in liver tissues (below the limit of detection), similar to the PBS control group. These results demonstrated that the Fiber_282–569_ subunit vaccine could provide complete protection for progeny chicks with maternal antibody (0/10 morbidity rate, 0/10 mortality rate, and 10/10 protection rate).

## 4. Discussion

### 4.1. Emergence of Hypervirulent FAdV-11 and the Critical Window of Susceptibility

This study confirms the emergence of virulent FAdV-11 strains, such as FAdV-11/HB2022/1, which caused 90–100% mortality in neonatal SPF chickens, representing a significant increase compared to historical reports of low pathogenicity [3,19,20]. This finding aligns with recent epidemiological trends of severe inclusion body hepatitis (IBH) outbreaks. A pivotal finding of this study is the strict age-dependent susceptibility: lethal infection occurred only in chicks challenged at 1 day of age, while chicks challenged at 7 days of age exhibited no mortality but developed mild disease and viral replication, and older chickens (≥14 days) were largely resistant.

This laboratory observation contrasts with field reports of IBH in older broilers (3–5 weeks of age). We hypothesize that this discrepancy may be attributed to three factors: (1) Differences in immune maturity versus field pressure [21]. While the immune system of 14-day-old SPF chickens can resist a single acute challenge, commercial chicks face continuous, high-dose environmental contamination that may overwhelm their developing immunity. (2) Co-infections [22]. Clinical outbreaks in older birds are often associated with immunosuppressive pathogens such as Chicken Anemia Virus (CAV) or Infectious Bursal Disease Virus (IBDV), which were absent in our SPF model. (3) Host genetic background [23]. The genetic background of commercial broilers may differ from that of SPF White Leghorns, thereby influencing susceptibility thresholds.

These findings indicate that although intrinsic age-dependent resistance exists in chickens, it can be compromised in commercial production environments, highlighting the critical need to protect chicks during their first week of life.

### 4.2. Rational Design of a Trimeric Fiber Subunit Antigen

The key to developing an effective subunit vaccine lies in presenting antigens in their native conformation. The homotrimeric state of the fiber protein is crucial for its function and for inducing neutralizing antibodies [12,24,25,26,27,28]. Unlike strategies using full-length proteins (which often form inclusion bodies), our design retained both the head domain (aa 361–569, associated with immune protection) and the shaft domain (aa 282–355).

The shaft domain is critical for trimerization via “knob-and-hole” interactions [11,24,25]. We hypothesized that monomeric fiber proteins would be structurally unstable and lack the conformational epitopes necessary to induce high-affinity neutralizing antibodies. Our SEC-MALS analysis provided definitive evidence. Compared with conventional molecular weight detection approaches including SDS-PAGE and standard size-exclusion chromatography (SEC), SEC-MALS presents unique advantages for absolute molecular weight measurement. Conventional boiling SDS-PAGE employs strong denaturing conditions that disrupt quaternary structure and predominantly resolves monomeric subunits. While specialized non-denaturing SDS-PAGE protocols may preserve oligomeric assemblies, standard SDS-PAGE cannot reliably confirm the native trimeric conformation of Fiber protein. Standard SEC calculates molecular weight via elution volume based on globular protein calibrants, which overestimates the molecular weight of elongated proteins such as Fiber due to their larger hydrodynamic radius and earlier elution [29,30,31]. Instead, SEC-MALS enables conformation- and elution time-independent absolute molecular weight quantification. Our measured molecular weight of 102.1 ± 4.25 kDa is highly consistent with the theoretical trimer mass (~106 kDa), directly verifying that the purified protein forms stable trimers in solution. Unlike studies merely adopting gel filtration or cross-linking tests, this accurate characterization confirms that the favorable bioactivity of the antigen originates from its properly folded trimeric conformation, which is likely the foundation of the potent immune response observed.

### 4.3. Maternal Antibody Transfer as an Effective Strategy to Bridge the Immunity Gap

The core achievement of this study is demonstrating that maternal antibodies (MatAb) can confer complete protection against lethal FAdV-11 infection. Conventional FAdV-11 vaccine evaluation models are flawed, as they often use older, inherently resistant chickens for challenge studies, potentially overestimating vaccine efficacy. By challenging 1-day-old neonatal chicks—the most susceptible population—this study adopted a more stringent and clinically relevant evaluation model. Similar models have also been applied in the prevention and control of other diseases as well as in vaccine development [32,33,34].

Our results show that maternal antibodies transferred from vaccinated breeders provided 100% protection, a finding consistent with reports in previous studies in the literature [13,35]. More importantly, protected chicks exhibited no detectable viral loads in liver tissues under the experimental challenge conditions. Challenged progeny chicks showed no clinical signs, no gross or histopathological lesions, and no detectable viral loads in the liver. This represents a fundamental distinction from vaccines that merely reduce viral load. However, since mucosal viral shedding was not evaluated in this study, we cannot formally conclude that this protection prevents viral shedding and horizontal transmission, which is essential for herd-level disease control and pathogen eradication [36,37,38,39]. This strategy effectively bridges the “immunity gap” during the first few days of life when chicks are most susceptible and their own immune systems are not yet mature enough to respond effectively to active immunization.

### 4.4. Limitations and Future Perspectives

While our findings are promising, several aspects warrant further investigation. First, this study only validated complete protection against lethal FAdV-11 challenge at 1 day of age, the peak point of neonatal susceptibility. The waning kinetics of maternally derived neutralizing IgY in growing chicks, and the exact duration of protective immunity beyond the immediate post-hatch period, were not systematically characterized in the present work. Longitudinal antibody monitoring and age-gradient challenge studies are still required to define the full protective window. This gap also strengthens the rationale for the proposed two-tiered immunization strategy: maternal antibody protection from breeder vaccination covers the high-risk neonatal window, and subsequent active subunit vaccination of broilers at 7–14 days of age can establish long-term active immunity as maternal antibodies naturally wane. Second, the breeder–progeny challenge design has inherent statistical limitations related to clustered observations. Progeny chicks within each group were derived from a fixed pool of 6 breeder hens, and offspring from the same hen share an identical maternal antibody source, meaning individual chicks are not fully statistically independent. Although we balanced sampling across all hens to minimize this bias, hen-to-hen variation in antibody transfer efficiency may still influence the results. Future studies with larger breeder hen cohorts and hierarchical statistical models that explicitly account for maternal clustering would strengthen the robustness of the findings. Third, this study was conducted in SPF chickens under controlled laboratory conditions; field trials in commercial broilers with varying genetic backgrounds and concurrent infections are necessary to validate efficacy under field conditions. Finally, while the fiber protein is a key antigen, evaluating the potential synergistic effects of combining fiber with other capsid proteins (e.g., penton base or hexon) could lead to the development of broader-spectrum vaccines.

This study focuses exclusively on protective efficacy against FAdV-11. Cross-protection against other FAdV serotypes (e.g., FAdV-4, FAdV-8b) was not evaluated. Given the serotype-specific nature of neutralizing epitopes on the Fiber protein, cross-protective capacity cannot be assumed. Future studies will be required to assess cross-reactive immunity and to develop multivalent fiber-based vaccines if broad serotype coverage is desired.

In our efficacy challenge experiment, intramuscular injection was selected as a stringent challenge model because our pathogenicity test demonstrated that IM inoculation consistently induces high-level mortality in neonatal chicks, providing a rigorous test for the protective capacity of maternal antibodies. Nevertheless, intramuscular challenge does not recapitulate natural mucosal exposure pathways under field conditions. Mucosal challenge models more representative of natural infection would be valuable in future work to better predict real-world vaccine performance.

Future studies should focus on a combined immunization strategy: breeder vaccination to protect neonates via maternal antibodies, followed by direct subunit vaccination of broilers at 7–14 days of age to establish active immunity as maternal antibodies wane. This two-tiered immunization strategy could provide seamless protection throughout the entire production cycle.

Our research group has established collaborative links with Qingdao Yebio Bioengineering Co., Ltd. for follow-up preclinical testing. Initial target markets would focus on major poultry-producing regions where hypervirulent FAdV-11 outbreaks are prevalent, including China and other Asian countries with intensive poultry production. Formal veterinary vaccine licensing will require comprehensive field safety and efficacy trials, production process validation, and regulatory documentation, which are beyond the scope of this basic research study and constitute future industrial development work.

## 5. Conclusions

In conclusion, we isolated a hypervirulent FAdV-11 strain (FAdV-11/HB2022/1) and demonstrated its high pathogenicity in neonatal chickens, characterized by severe age-dependent mortality. We successfully developed a soluble, trimeric Fiber subunit vaccine (Fiber_282–569_) using a rational truncation strategy in an *E. coli* expression system. Crucially, we proved that immunization of breeder hens with this subunit vaccine induces high levels of neutralizing antibodies that are vertically transferred to progeny. These maternal antibodies provided complete protection against lethal FAdV-11 challenge in 1-day-old chicks at the peak of neonatal susceptibility under laboratory conditions, inhibiting viral replication and abrogating liver pathology. This study validates the “breeder vaccination–progeny protection” strategy as a safe and effective immunological framework for controlling FAdV-11 under laboratory conditions, with potential practical value for the poultry industry following further safety assessment. Formal cost-effectiveness analyses and field validation in commercial production systems will be required to determine its real-world economic and operational viability.

## Figures and Tables

**Table 1:** Homology modeling results of FAdV-11 Fiber.

Model ID	Template	Modeling Quality Assessment	Sequence Similarity	Covered Sequence Sites	Template Information	Oligomers	Remarks
1	7w83.1.A	0.70 ± 0.05	38.38%	361–569	FAdV-4 fiber-2 protein	Homotrimer	Head
2	3s6x.1.A	0.38 ± 0.07	22.73%	282–355	Outer capsid protein sigma-1	Homotrimer	Tail-shaft
3	6gap.1.A	0.44 ± 0.06	17.46%	97–161	Outer capsid protein sigma-1	Homotrimer	Tail-shaft
4	6gap.1.A	0.36 ± 0.06	16.67%	166–225	Outer capsid protein sigma-1	Homotrimer	Tail-shaft
5	6gap.1.A	0.39 ± 0.08	15.00%	320–359	Outer capsid protein sigma-1	Homotrimer	Tail-shaft

## Data Availability

The original contributions presented in this study are included in the article. Further inquiries can be directed to the corresponding author.

## Supplementary Materials

The following supporting information can be downloaded at: https://www.mdpi.com/article/10.3390/vetsci13090928/s1, Figure S1: Original full-length Western blot image with molecular weight markers. Table S1: Reference FAdV strains used for hexon gene-based phylogenetic analysis. The table includes 29 reference strains spanning FAdV species A–E, with strain names, serotype classification, GenBank accession numbers and corresponding references. All sequences are fully consistent with those used for phylogenetic tree construction in Figure 1A. File S1: Completed ARRIVE 2.0 Author Checklist. The checklist for reporting animal experiments in accordance with the ARRIVE 2.0 guidelines. File S2: Supporting official documents. Signed conflict-of-interest disclosure form and the official animal ethics approval document (approval No. 2021-01-002) issued by the Animal Ethics Committee of Yangtze University.

## Abbreviations

FAdV, fowl adenovirus; IBH, inclusion body hepatitis; TCID_50_, 50% tissue culture infectious dose; SPF, specific-pathogen-free; LMH, leghorn male hepatoma cell line; HE, hematoxylin–eosin staining; qPCR, quantitative real-time polymerase chain reaction; SEC-MALS, size-exclusion chromatography coupled with multi-angle light scattering; IgY, immunoglobulin Y; IFA, indirect immunofluorescence assay; MatAb, maternal antibody; SDS-PAGE, sodium dodecyl sulfate–polyacrylamide gel electrophoresis; TMB, 3,3′,5,5′-Tetramethylbenzidine; CPE, cytopathic effect.
