# Supplementary material for: Maternal Antibody Transfer of a Fiber-Based Subunit Vaccine Confers Complete Protection Against Fowl Adenovirus Serotype 11 in Neonatal Chickens
Source: Vet Sci. 2026 Sep 9;13(9):928. doi: 10.3390/vetsci13090928 (PMC13611989; doi:10.3390/vetsci13090928)
Supplement: Supplementary file 1 [file vetsci-13-00928-s001.zip › Figure S1.pdf]

Figure S1

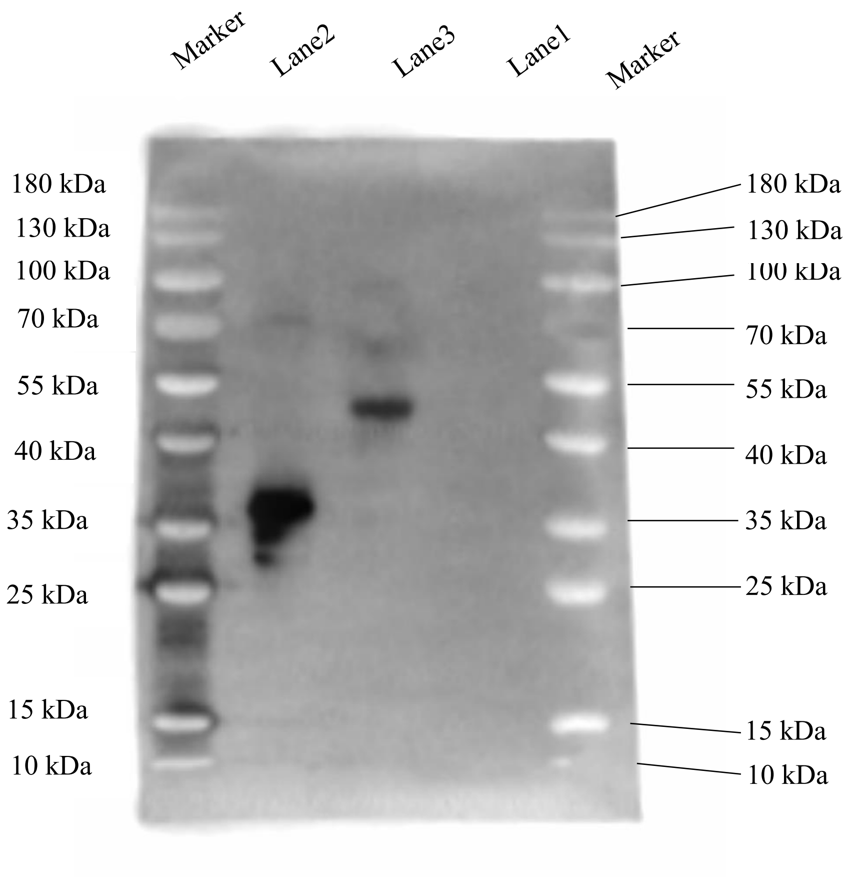

Marker
Lane2
Lane3
Lane1
Marker
180 kDa
130 kDa
100 kDa
70 kDa
55 kDa
40 kDa
35 kDa
25 kDa
15 kDa
10 kDa
180 kDa
130 kDa
100 kDa
70 kDa
55 kDa
40 kDa
35 kDa
25 kDa
15 kDa
10 kDa
