# Supplementary material for: Maternal Antibody Transfer of a Fiber-Based Subunit Vaccine Confers Complete Protection Against Fowl Adenovirus Serotype 11 in Neonatal Chickens
Source: Vet Sci. 2026 Sep 9;13(9):928. doi: 10.3390/vetsci13090928 (PMC13611989; doi:10.3390/vetsci13090928)
Supplement: Supplementary file 1 [file vetsci-13-00928-s001.zip › File S2.pdf]

MDPI

Multidisciplinary
Digital Publishing
Institute

MDPI AG
St Alban-Anlage 66
CH–4052 Basel
Switzerland

Tel. +41 61 683 77 34
Fax +41 61 302 89 18
www.mdpi.com

Disclosure of Potential Conflicts of Interest

All authors must disclose all relationships or interests that could inappropriately influence or bias their work. Although the presence of a relationship or activity does not always indicate a problematic influence on a paper's content, perceptions of conflict can erode trust in science as much as actual conflicts of interest. Ultimately, readers must be able to make their own judgments regarding whether an author's relationships and activities are pertinent to a paper's content. These judgments require transparent disclosures. An author's complete disclosure demonstrates a commitment to transparency and helps to maintain trust in the scientific process.

The corresponding author collects the conflict of interest disclosure from all authors. The corresponding author must include a summary statement in the manuscript in a separate section "Conflicts of Interest" placed just before the reference list. The statement should reflect all the collected potential conflict of interest disclosures in the form.

Journal Name:	Veterinary Sciences
Manuscript ID (if known):	vetsci-4537392
Manuscript Title:	Maternal Antibody Transfer of a Fiber Based Subunit Vaccine Confers Complete Protection against Fowl Adenovirus Serotype 11 in Neonatal Chickens
Authors:	Guopan Li, Sirong Gao, Xiaoyang Ai, Jun Rong, Disi Chen, Lianshen Cai, Weiwei Guo
Corresponding Author(s):	Xiaoyang Ai; Jun Rong

Financial interests

These may include but are not limited to membership, employment, consultancies, stocks/shares ownership; honoraria; grants or other funding; paid expert testimony and patent-licensing arrangements. Some examples are as follows:

- An author has received or expects to receive research grants from funding agencies (please provide details regarding the research funder and the grant number)
- An author has received support from commercial sources of funding by companies that sell drugs, medical devices, or provide medical services
- An author has received honoraria for speaking at symposia
- An author holds a position on advisory boards
- An author is an inventor of any planned, pending, or awarded patent on this work (please provide detailed information such as regarding the patent applicant, name of inventors, application number, status of application, specific aspects of manuscripts covered in the patent application)
- An author is an employee of an organization or company

Non-financial interests

These may include but are not limited to personal relationships or competing interests directly or indirectly tied to this research, or professional interests or personal beliefs that may influence your research.

The authors whose names are listed immediately below certify that they have NO conflicts of interest to declare:

Guopan Li, Sirong Gao, Xiaoyang Ai, Disi Chen

The authors whose names are listed immediately below report the following conflicts of interest related to the work under consideration:

Jun Rong, Lianshen Cai, Weiwei Guo: They are employees of Qingdao Yebio Bioengineering Co., Ltd.

This statement is signed by all the authors to indicate agreement that the above information is true and correct (*a photocopy of this form may be used if there are more than 10 authors*):

Author's name (typed)	Author's signature	Date
Guopan Li	李国攀	2026.8.18
Sirong Gao	高思蓉	2026.8.18
Xiaoyang Ai	艾笑扬	2026.8.18
Jun Rong	荣俊	2026.8.18
Disi Chen	陈[illegible]斯	2026.8.18.
Lianshen Cai	蔡联燊	2026.8.18
Weiwei Guo	郭伟伟	2026.8.18

No. 2021-01-002

Animal Experimental Ethical Inspection Form

Dear Editorial Board,

In the animal experimental research part of the manuscript entitled *"Maternal Antibody Transfer of a Fiber-Based Subunit Vaccine Confers Complete Protection against Fowl Adenovirus Serotype 11 in Neonatal Chickens"*, the authors abide by the rules of animal experimental ethics, accept the supervision and inspection of the animal experimental ethics committee, and accept the punishment if any infringement.

During the research process, the following points must be observed:

1. On the premise of not affecting the scientific nature and comparability of experimental results, high-grade animal should be replaced with low-grade animal and cell culture should be used instead of whole animal.

2. The quantity of animals should be reduced by improving the study design or using high quality animals.

3. The study design should be reasonable to protect experimental animals.

Discussed by all members of Ethics Committee of Health Science Center, Yangtze University, the animal experimental research part of the manuscript met the requirements of medical ethics, which was accepted with unanimous approval and agreed to be published.

Ethics Committee of Health Science Center, Yangtze University
(长江大学医学部伦理委员会)
January 5, 2021
